# Differential Effects of PTH (1-34), PTHrP (1-36), and Abaloparatide on the Murine Osteoblast Transcriptome

**DOI:** 10.1210/jendso/bvad156

**Published:** 2023-12-13

**Authors:** Michael J Mosca, Zhiming He, Florante R Ricarte, Carole Le Henaff, Nicola C Partridge

**Affiliations:** Department of Molecular Pathobiology, New York University College of Dentistry, New York, NY 10010, USA; Vilcek Institute of Graduate Biomedical Sciences, New York University School of Medicine, New York, NY 10016, USA; Department of Molecular Pathobiology, New York University College of Dentistry, New York, NY 10010, USA; Department of Molecular Pathobiology, New York University College of Dentistry, New York, NY 10010, USA; Department of Molecular Pathobiology, New York University College of Dentistry, New York, NY 10010, USA; Department of Molecular Pathobiology, New York University College of Dentistry, New York, NY 10010, USA

**Keywords:** osteoblast, parathyroid hormone (PTH), osteoporosis, abaloparatide, RNA-Seq, signal transduction

## Abstract

Teriparatide (PTH (1-34)), PTHrP (1-36), and abaloparatide (ABL) have been used for the treatment of osteoporosis, but their efficacy long term is significantly limited. The 3 peptides exert time- and dose-dependent differential responses in osteoblasts, leading us to hypothesize they may also differentially modulate the osteoblast transcriptome. Treatment of mouse calvarial osteoblasts with 1 nM of the peptides for 4 hours results in RNA sequencing data with PTH (1-34) regulating 367 genes, including 194 unique genes; PTHrP (1-36) regulating 117 genes, including 15 unique genes; and ABL regulating 179 genes, including 20 unique genes. There were 83 genes shared among all 3 peptides. Gene ontology analyses showed similarities in Wnt signaling, cAMP-mediated signaling, ossification, but differences in morphogenesis of a branching structure in biological processes; receptor ligand activity, transcription factor activity, and cytokine receptor/binding activity in molecular functions. The peptides increased *Vdr*, *Cited1*, and *Pde10a* messenger RNAs (mRNAs) in a pattern similar to *Rankl*, that is, PTH (1-34) greater than ABL greater than PTHrP (1-36). mRNA abundance of other genes, including *Wnt4*, *Wnt7*, *Wnt11*, *Sfrp4*, *Dkk1*, *Kcnk10*, *Hdac4*, *Epn3*, *Tcf7*, *Crem*, *Fzd5*, *Ppp2r2a*, and *Dvl3*, showed that some genes were regulated similarly by all 3 peptides; others were not. Finally, small interfering RNA knockdowns of SIK1/2/3 and CRTC1/2/3 in PTH (1-34)–treated cells revealed that *Vdr* and *Wnt4* genes are regulated by salt-inducible kinases (SIKs) and CREB-regulated transcriptional coactivators (CRTCs), while others are not. Although many studies have examined PTH signaling in the osteoblast/osteocyte, ours is the first to compare the global effects of these peptides on the osteoblast transcriptome or to analyze the roles of the SIKs and CRTCs.

Teriparatide (PTH (1-34)), a recombinant form of PTH, was the first osteoanabolic therapeutic to be approved by the US Food and Drug Administration [1, 2]. Abaloparatide (ABL), an analogue of PTH-related protein (PTHrP (1-34), gene name *PTHLH*), became the second Food and Drug Administration–approved osteoanabolic for treatment of osteoporosis and has been shown to be somewhat more effective in producing osteoanabolic outcomes compared with teriparatide; ABL resulted in higher bone mineral density in the femurs of osteoporotic, postmenopausal women compared with teriparatide [3, 4, 5]. It has also been shown that serum CTX levels, a marker for bone resorption, were lower in patients treated with ABL compared with teriparatide, suggesting it has a lesser tendency to promote deleterious effects [5]. Despite this, neither ABL nor teriparatide has been able to overcome the anabolic window preventing the long-term efficacy of these treatments for osteoporotic patients [6].

PTH, PTHrP, and ABL all bind the same G protein–coupled receptor, PTH receptor type 1 (PTHR1, gene name *PTH1R*) [7]. One study found that PTH (1-34), PTHrP (1-36), and ABL act through 2 PTHR1 conformations named R^O^ and RG. Binding to R^O^ results in prolonged signaling, which is thought to lead to comparatively more bone resorption, while binding to RG is thought to result in more osteoanabolic signaling [8]. The study determined that ABL binds with greater selectivity to RG and concluded this represents a plausible explanation for the favorable anabolic effects of ABL treatment reported on bone compared with PTH (1-34). The differences observed in this study of PTH (1-34), PTHrP (1-36), and ABL binding to PTHR1 suggest these findings may be reflected by differential signaling events downstream of PTHR1. However, there is a paucity of data that have determined differences in signaling from PTH (1-34), PTHrP (1-36), and ABL in osteoblast lineages.

There are a variety of signaling cascades that are stimulated on PTHR1 ligand binding. The G_Sα_/cAMP/PKA pathway accounts for most of PTHR1 signaling, and this is purported to mediate the anabolic response to PTH as well as the catabolic response [9, 10, 11, 12]. Recent work from our laboratory examined if PTH (1-34), PTHrP (1-36), and ABL treatment would result in unique stimulatory effects with respect to cyclic adenosine monophosphate (cAMP) production and known downstream effectors of this pathway, the salt-inducible kinases (SIKs) and CREB-regulated transcriptional coactivators (CRTCs) [13]. We found that in primary murine calvarial osteoblasts, PTHrP (1-36) and ABL result in a significantly lower cAMP response compared with PTH (1-34). Downstream of this signaling cascade, time course and dose response analyses showed similar relative differences in protein kinase A (PKA) activation and the phosphorylation of cAMP response element binding protein (CREB). However, quantitative reverse-transcription PCR (qRT-PCR) of known osteoblastic genes found several genes were similarly regulated such as the Wnt inhibitor, *Sost*, while *c-Fos* and *Rankl* (*Tnfsf11*) were differentially regulated in time- and dose-dependent manners. It is interesting that another research group found that ABL gave a higher stimulation of cAMP in MC3T3-E1 cells than teriparatide and slightly higher osteoanabolic effect in female mice, and the same group also showed that ABL seemed to have a protective effect on cortical bone loss [14, 15].

Since we determined that these peptides differentially exert time- and dose-dependent responses in the osteoblast, particularly on the cAMP/PKA/*Fos* or *Rankl* axes, we hypothesized that these changes reflect the ability of PTH (1-34), PTHrP (1-36), and ABL to differentially modulate the osteoblast transcriptome. Many studies have examined PTH and PTHrP regulation of gene expression in stromal cells/osteoblasts/osteocytes but none to date have compared the global effects of these 3 peptides on the osteoblast transcriptome [16, 17, 18, 19]. In this study, RNA sequencing (RNA-Seq) was performed on primary calvarial murine osteoblasts treated with PTH (1-34), PTHrP (1-36), and ABL by repeating the conditions under which we observed differences in *Rankl* expression to compare their global effects on the osteoblast transcriptome and gene ontology [13]. Select findings were confirmed via qRT-PCR of additional cultured samples of mouse calvarial osteoblasts, treated with 1 nM of PTH (1-34), PTHrP (1-36), or ABL for 4 hours prior to harvest. Lastly, analyses were performed in which SIK1, SIK2, SIK3, CRTC1, CRTC2, and CRTC3 were knocked down in cells treated with 10 nM of PTH (1-34) to examine possible intertwined/separate cAMP/SIK/CRTC-dependent regulation of a number of these genes.

We hypothesized that the differences among these peptides published previously on their binding affinities, clinical outcomes, and downstream expression of anabolic/catabolic effectors would be reflected at the transcriptional level and that unique differences would be elicited by each peptide, allowing us to determine new signaling biases of interest. If these peptides lead to differential expression of osteoblastic or unknown cascades/genes, then this process may provide key insights for future studies to better understand the osteoanabolic and catabolic effects of PTHR1-derived treatments so more effective therapeutics can be developed to treat osteoporosis.

## Materials and Methods

### Peptides and Chemicals

Rat PTH 1-34 was purchased from Bachem. PTHrP 1-36 and ABL were synthesized by the Peptide/Protein Core Facility at the Massachusetts General Hospital. All PTH (1-34), PTHrP (1-36), and ABL peptide sequences were confirmed and analyzed for purity and degradation by the NYU Grossman School of Medicine Mass Spectrometry Core Facility. All peptides were dissolved in 10 mM acetic acid. Ascorbic acid was purchased from Sigma. Collagenase A was purchased from Worthington Biochemical Corporation.

### Cell Culture

Primary mouse calvarial osteoblasts were harvested from C57Bl/6J wild-type mice aged 2 to 3 days post natal. Mice were euthanized with ketamine (0.25 mg/pup) and xylazine (0.025 mg/pup). All procedures with mice were performed in accordance with an approved protocol of the Institutional Animal Care and Use Committee of New York University Grossman School of Medicine. Calvariae were digested in 1 mg/mL collagenase A at 37 °C by 5 sequential digestions, and cells from digests 3 to 5 were collected and plated at a density of 6.4 × 10^3^ cells/cm^2^ in αMEM supplemented with 10% fetal bovine serum, 100 units/mL penicillin, 100 μg/mL streptomycin, and 0.25 μg/mL of amphotericin B. After reaching confluence, osteogenic medium (50 μg/mL ascorbic acid) was added for 5 days to allow osteoblastic differentiation. Prior to harvest, osteoblasts were treated with 1 or 10 nM of the peptides for 4 hours (n = 3 per group). Prior to treatment, cells were serum-starved with 0.1% fetal bovine serum for 16 hours.

### Small Interfering RNA Knockdowns

Primary mouse calvarial osteoblasts were harvested from C57Bl/6J wild-type mice aged 2 to 3 days post natal and plated using the same protocol as described earlier until they reached 70% to 80% confluence. The cells were given small interfering RNAs (siRNAs) in Lipofectamine RNAiMAX for 48 hours in differentiation medium. The manufacturer's protocol was followed so that 40 pmol of siRNA was used per well (6 well plates) with 8 µL of Lipofectamine. After 48 hours of siRNA transfection, the cells were then treated with or without PTH (1-34) at 10 nM for 4 hours and RNA was harvested using TRIzol reagent. Additional samples were collected for protein analysis after isolation with radioimmunoprecipitation assay buffer. Confirmation of siRNA knockdowns was conducted with qRT-PCR examining messenger RNAs (mRNAs) and Western blots for proteins (data not shown; under consideration in a separate manuscript). Relative expression of *Rpl13a*, *Alpl*, and *Col1a1* were examined to determine if SIK or CRTC knockdowns affected cell viability and general processes. In general, cells tolerated all knockdowns well and did not die or alter their housekeeping or cell-specific genes significantly (data not shown; under consideration in a separate manuscript).

### RNA Sequencing

Total RNA was isolated from cells by using TRIzol reagent (Thermo Scientific) and purified with an RNeasy mini kit from Qiagen. Prior to RNA-seq, RNA integrity was assessed with an Agilent 2100 Bioanalyzer and the best-quality triplicate samples were chosen for the subsequent analyses. The RNA-seq libraries were constructed using the Illumina TruSeq Stranded Total RNA library prep kit with Ribozero Gold. Sequencing was carried out with an Illumina HiSeq 2500 system with paired-end 100 bp reads at the Genome Technology Center of the NYU Grossman School of Medicine. The quality of raw data was checked by FastQC (v. 0.11.9), and the read counts were quantified using Salmon (v. 1.7.0) against the GRCm38/mm10 mouse transcriptome reference (UCSC) database [20]. Pairwise differential expression analysis was performed by the DESeq2 R/Bioconductor package (v. 1.34.0) [21]. The data have been submitted to GEO and have the accession number GSE240235. Where gene expression was found to be significantly above ±1.0 log_2_ fold change (FC) after treatments, these genes were imputed in the DAVID Bioinformatics Database NIAID/NIH for Gene Ontology (GO) analyses [22].

### Quantitative Reverse-Transcription Polymerase Chain Reaction

Total RNA was extracted using TRIzol (Sigma). Complementary DNA was synthesized from 1 μg of total RNA using a TaqMan reverse-transcription kit (Applied Biosystems) with hexamer primers following the protocol described by the manufacturer. Gene expression levels were measured using SYBR Green PCR Reagents (Applied Biosystems). The quantity of mRNA was calculated by normalizing the threshold cycle value of specific genes to the cycle value of the housekeeping genes *β-actin* and/or Ribosomal protein L13a (*Rpl13a*).

### Statistics

Statistical differences were analyzed either by *t* test or one-way analysis of variance using IBM SPSS (v24). Tukey tests were then performed to determine which groups in the sample differed significantly from one another. Results are expressed as mean ± SD, and a *P* value less than .05 was considered statistically significant comparing treatment groups.

## Results

### Global Expression Profiles for Parathyroid Hormone (1-34), Parathyroid Hormone–Related Protein (1-36), and Abaloparatide

**Figure 1.**
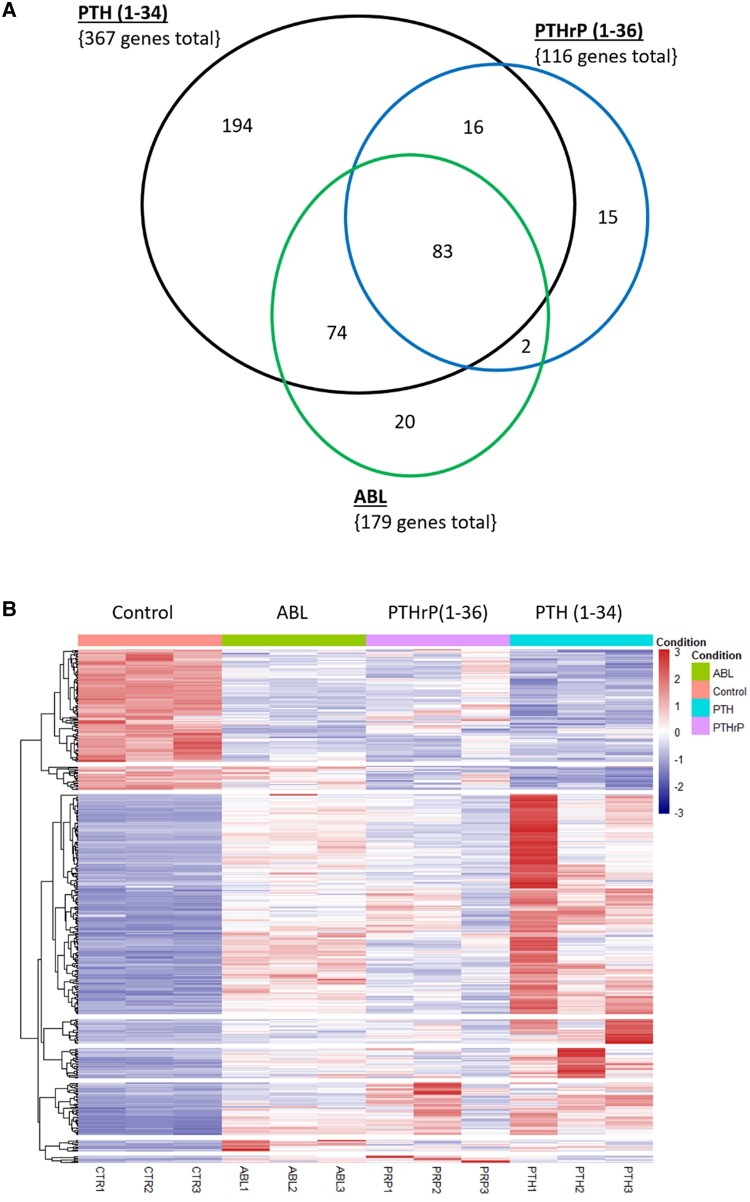
Venn diagram and heat map of parathyroid hormone (PTH) (1-34), PTH-related protein (PTHrP) (1-36), and abaloparatide (ABL) treatment on the osteoblast transcriptome. Primary mouse calvarial osteoblasts were treated with 1 nM of these peptides for 4 hours and gene enrichment analysis of RNA-sequencing data was performed. Gene lists were selected with log_2_ fold change (FC) greater than or equal to 1; false discovery rate (FDR) less than 0.05 compared with the vehicle controls. A, PTH (1-34) regulated 367 genes, 194 were unique; PTHrP (1-36) regulated 116 genes, 15 were unique; ABL regulated 179 genes, 20 were unique. There were 74 genes shared only among PTH(1-34) and ABL; 16 genes shared only among PTH (1-34) and PTHrP; and 83 genes shared among all 3 peptides. B, All genes in the Venn diagram were subjected to heat map analysis. Red represents transcript upregulation; blue represents transcript downregulation. Analysis is relative to control samples. Genes were selected if they had a log_2_ FC greater than or equal to 1 and had an FDR less than 0.05.

**Figure 2.**
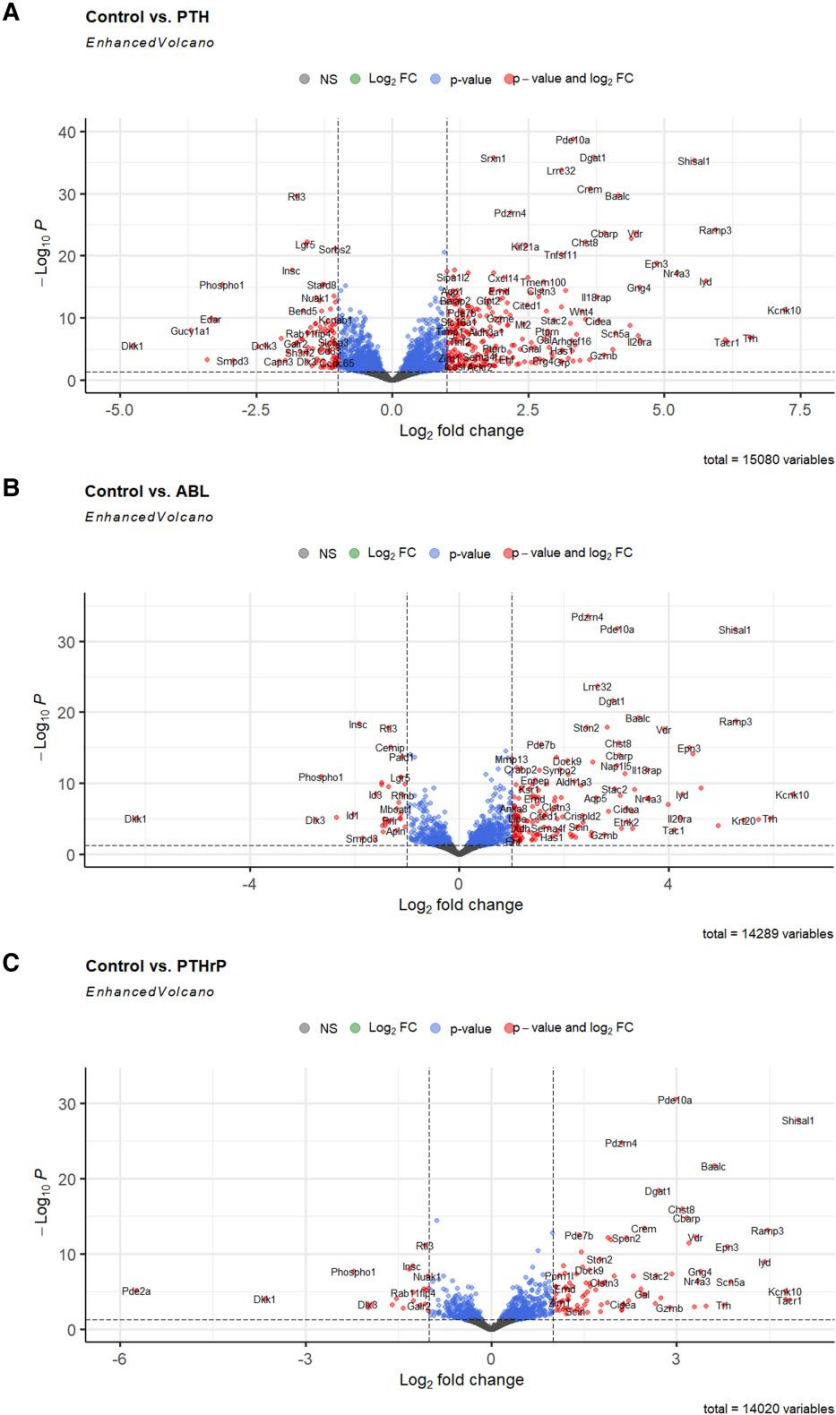
Volcano plots of parathyroid hormone (PTH) (1-34), PTH-related protein (PTHrP) (1-36), and abaloparatide (ABL) regulation of the osteoblast transcriptome. Primary mouse calvarial osteoblasts were treated with 1 nM of these peptides for 4 hours and gene enrichment analysis of RNA-sequencing data was performed. Genes were selected if they had a log2 fold change (FC) greater than or equal to 1 and had a false discovery rate (FDR) less than 0.05. A, PTH (1-34) vs control; B, ABL vs control; and C, PTHrP (1-36) vs control. Genes that are red on the graph are significantly regulated and have a log2 FC greater than or equal to 1; genes represented by blue were significantly regulated but did not pass the FC threshold, and genes represented by gray had no significant changes compared to control with either selection.

Genes were selected if they had a log_2_ FC greater than or equal to 1 and a false discovery rate (FDR) less than 0.05. RNA-seq revealed that PTH (1-34) regulated 367 genes, 194 were unique; PTHrP (1-36) regulated 116 genes, 15 were unique; and ABL regulated 179 genes, 20 were unique. There were 74 genes shared only among PTH (1-34) and ABL; 16 genes shared only among PTH (1-34) and PTHrP; and notably 83 genes shared among all 3 peptides. These data were analyzed and compiled into Venn diagrams (Fig. 1A), heat maps comparing peptides (Fig. 1B), and volcano plots (Fig. 2). The heat maps show there is a complete change in gene expression with PTH (1-34) and that this peptide causes greater quantitative effects than the other peptides, with PTHrP causing the least effects. The volcano plots further illustrate this and show some of the specific genes regulated and that many of the highest regulated genes are the same for the 3 peptides but often differ in degree of regulation indicating a general similarity in action.

### Pathway Analyses of Parathyroid Hormone (1-34), Parathyroid Hormone–Related Protein (1-36), and Abaloparatide Treatment on the Osteoblast Transcriptome

**Figure 3.**
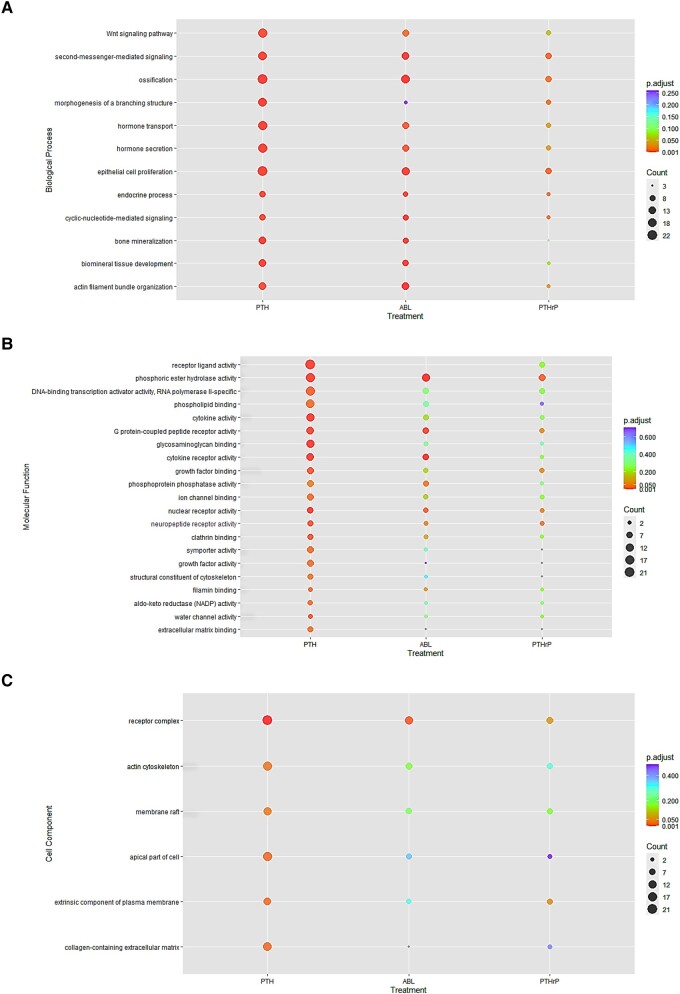
Pathway specific comparison of parathyroid hormone (PTH) (1-34), abaloparatide (ABL), and PTH-related protein (PTHrP) (1-36) treatment on osteoblast transcriptome pathways. Primary mouse calvarial osteoblasts were treated with 1 nM of these peptides for 4 hours and gene enrichment analysis of RNA-sequencing data was performed. If gene expression was found to be above ±1.0 log2 FC genes were imputed in DAVID Bioinformatics Database NIAID/NIH for Gene Ontology (GO) analysis; data for control vs peptide were graphed via R. A, Biological processes; B, molecular functions; and C, cell components.

Analyses of RNA-seq data were performed using GO to determine pathway-specific trends from differentially expressed genes according to each peptide treatment (Fig. 3). These were analyzed via R to identify the gene ontology biological processes, molecular functions, and cellular components affected by the 3 peptides. In Fig. 3 the size of the bubbles corresponds to the number of genes each peptide regulated per category, and the color represents the level of significance. With respect to biological processes, PTH (1-34) and ABL have similarly high levels of significance for all categories except for “morphogenesis of branching structures,” where ABL does not significantly regulate this process, while PTHrP (1-36) and PTH (1-34) showed more common regulation of genes of this process. PTH (1-34) and ABL show an almost identical pattern otherwise, and PTHrP (1-36) regulates these processes differently when compared with either of these peptides. Some processes were regulated by all three, some by just two. Notably, PTH (1-34) and ABL similarly regulated genes of “bone mineralization,” “biomineral tissue development,” and “actin filament bundle organization,” while PTHrP (1-36) did not regulate these processes significantly. Many molecular functions were highly regulated by PTH (1-34), for example, receptor ligand activity, transcription factor activity, phospholipid binding, and cytokine and receptor activity. Several were similarly regulated by all 3 peptides: phosphoric ester hydrolase activity, G protein receptor activity, and nuclear receptor activity. Likewise, PTH (1-34) generally gave the greatest and most significant regulation of all the cell components regulated, such as receptor complex, membrane rafts, and collagen-containing extracellular matrix. PTHrP yielded the least effects for both molecular functions and cell components.

### Examination of Genes of Interest With RNA Sequencing Data and Quantitative Reverse-Transcription Polymerase Chain Reaction Verification

Based on these data, we selected several genes that were highly upregulated or downregulated to examine more closely and to verify the RNA-seq data with qRT-PCR in separate calvarial osteoblast preparations. The genes included 1) Cbp/P300 interacting transactivator with Glu/Asp rich carboxy-terminal domain 1 (*Cited1*), 2) vitamin D receptor (*Vdr*), 3) phosphodiesterase 10A (*Pde10a*), 4) Wnt Family Member 11 (*Wnt11*), 5) Secreted frizzled related protein 4 (*Sfrp4*), 6) cAMP responsive element modulator (*Crem*), 7) Epsin 3 (*Epn3*), 8) Histone deacetylase 4 *(Hdac4*), 9) protein phosphatase 2 regulatory subunit B alpha (*Ppp2r2a*), 10) Disheveled segment polarity protein 3 (*Dvl3*), 11) *Wnt4*, 12) *Wnt7b*, 13) Dickkopf1 (*Dkk1*), 14) Frizzled class receptor 5 (*Fzd5*), 15) transcription factor *7* (*T-cell specific, HMG-box*) (*Tcf7*), and 16) potassium 2 pore domain channel subfamily K member 10 (*Kcnk10*). RNA-seq log_2_ FCs/*P*adj values are shown in Table 1.

**Table 1.** Regulation of genes of interest by RNA sequencing after parathyroid hormone (1-34), abaloparatide, and parathyroid hormone–related protein (1-36) treatment in differentiating calvarial osteoblasts.

Gene ID	Gene name	PTH (1-34)		ABL		PTHrP (1-36)	
		Log2 FC	*P*adj	Log2 FC	*P*adj	Log2 FC	*P*adj
***Cited1***	Cbp/P300 interacting transactivator 1	2.48	6.92E-13	1.61	3.17E-06	1.56	1.05E-05
***Vdr***	Vitamin D receptor	4.47	2.31E-24	3.90	2.25E-18	3.31	5.29E-13
***Pde10a***	Phosphodiesterase 10A	3.33	1.60E-39	3.02	1.63E-32	2.97	3.04E-31
***Wnt11***	Wnt family member 11	1.53	5.19E-06	1.15	5.49E-04	0.14	3.32E-01
***Sfrp4***	Secreted frizzled related protein 4	1.14	3.00E-07	1.05	3.77E-06	0.36	4.09E-02
***Crem***	cAMP responsive element modulator	3.64	1.48E-31	2.83	1.30E-18	2.48	3.55E-14
***Epn3***	Epsin 3	4.86	1.75E-19	4.40	9.08E-16	3.82	9.07E-12
***Hdac4***	Histone deacetylase 4	1.30	7.33E-12	1.20	3.51E-10	1.17	3.23E-09
***Ppp2r2a***	Protein phosphatase 2 regulatory subunit B alpha	0.21	2.88E-01	0.01	9.43E-01	0.39	5.23E-02
***Dvl3***	Disheveled segment polarity protein 3	0.09	6.42E-01	0.03	8.17E-01	0.76	1.14E-02
***Wnt4***	Wnt family member 4	3.49	7.44E-12	3.08	5.26E-09	1.87	2.48E-04
***Wnt7b***	Wnt family member 7b	0.27	1.73E-01	0.40	6.22E-02	0.11	4.09E-01
***Dkk1***	Dickkopf WNT signaling pathway inhibitor 1	−4.77	2.31E-06	−6.18	6.84E-06	−3.65	8.23E-05
***Fzd5***	Frizzled class receptor 5	−1.28	1.32E-06	−0.81	1.83E-03	−0.15	2.90E-01
***Tcf7***	Transcription factor 7 (T-cell–specific, HMG-box)	−0.57	3.42E-02	−0.13	3.44E-01	−0.21	1.53E-01
***Kcnk10***	Potassium 2 pore domain channel subfamily K member 10	7.22	4.30E-12	6.36	3.22E-09	4.76	8.25E-06

Primary mouse calvarial osteoblasts were treated with 1 nM of these peptides for 4 hours and gene enrichment analysis of RNA-sequencing data was performed. Gene lists were selected with log_2_ FC greater than or equal to 1; false discovery rate less than 0.05 compared with vehicle controls. (+) log_2_ FC represents upregulation compared to control; (−) log_2_ FC represents downregulation compared to control.

Abbreviations: ABL, abaloparatide; cAMP, cyclic adenosine monophosphate; FC, fold change; PTH, parathyroid hormone; PTHrP, parathyroid hormone–related protein.

**Figure 4.**
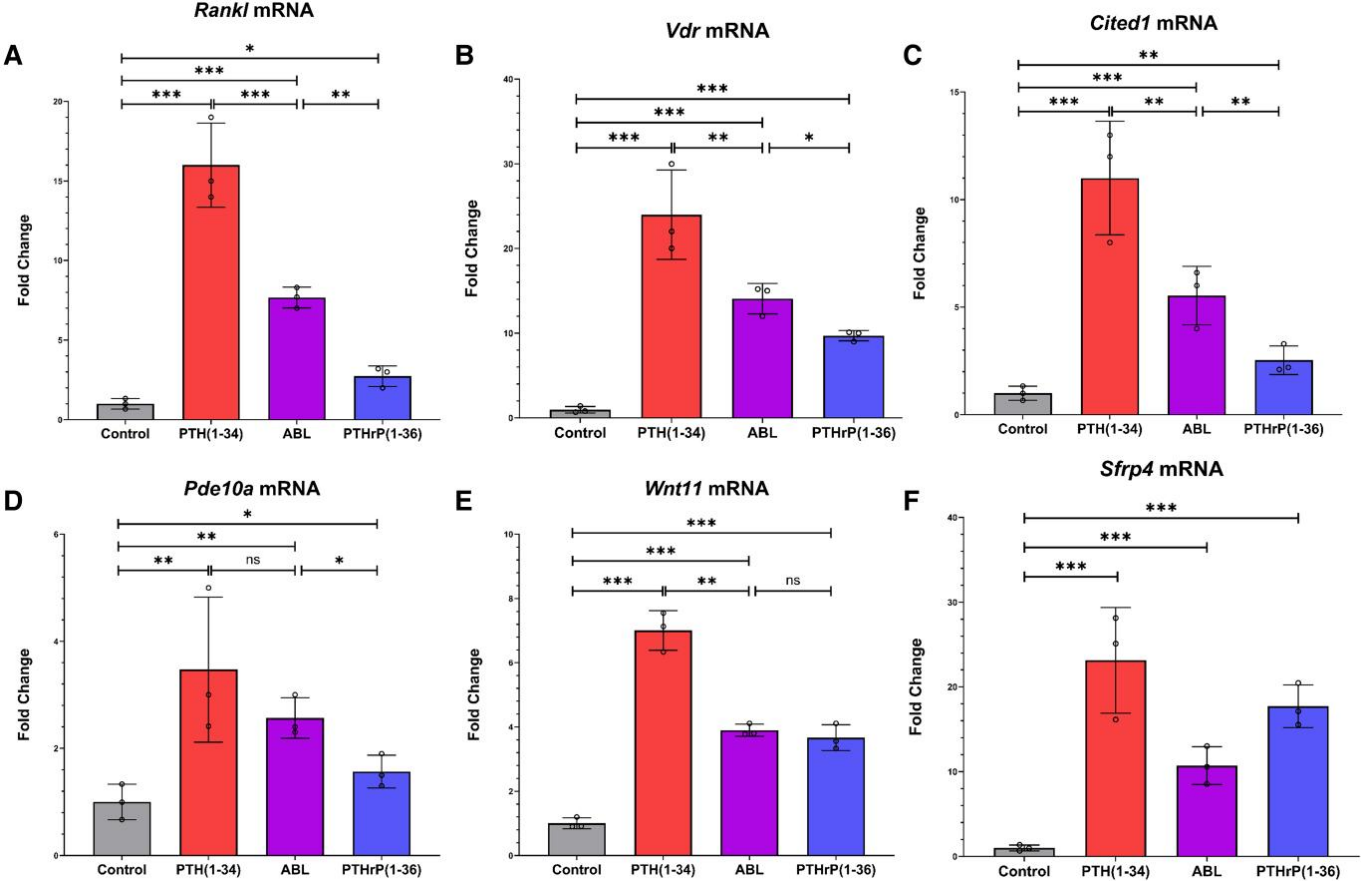
Parathyroid hormone (PTH) (1-34), PTH-related protein (PTHrP) (1-36), and abaloparatide (ABL) modulate expression of *Vdr*, *Cited1*, *Pde10a*, *Wnt11*, and *Sfrp4* messenger RNA (mRNA) with a similar pattern to *Rankl*. Primary differentiating calvarial osteoblasts were treated with 1 nM of PTH (1-34), PTHrP (1-36), or ABL for 4 hours prior to harvest, followed by quantitative reverse-transcription polymerase chain reaction for *Vdr*, *Cited1*, *Pde10a*, *Wnt11*, *Sfrp4*, and *Rankl* mRNAs. All data are expressed relative to the housekeeping gene *Rpl13a* and represent mean ± SD of n = 3 independent experiments. *P less than .05; **P less than .01; ***P less than .001; ns, not significantly different.

qRT-PCR data confirmed *Vdr*, *Cited1*, *Pde10a*, and *Wnt11* mRNAs followed the reported pattern of expression profiling of *Rankl* in response to these peptides: PTHrP (1-36) and ABL elicit a moderate increase in *Rankl* compared with control with PTHrP (1-36), being in most cases significantly lower than ABL and PTH (1-34) eliciting the greatest increase in expression of these genes compared with control (Fig 4). *Sfrp4* closely mimicked this expression pattern but with one minor difference in just the qPCR data: PTHrP (1-36) resulted in higher mRNA expression than ABL but both were still lower than PTH (1-34) treatment.

**Figure 5.**
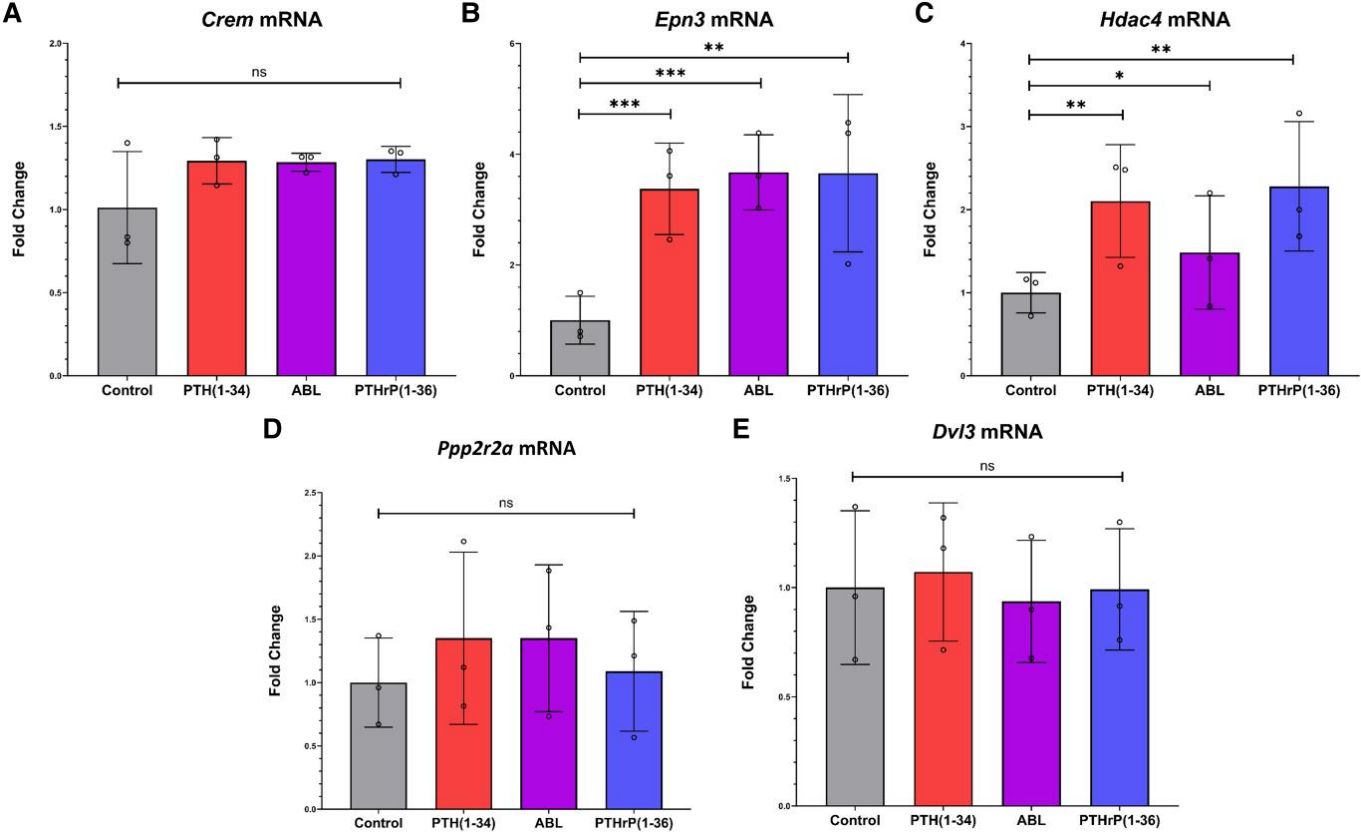
Parathyroid hormone (PTH) (1-34), PTH-related protein (PTHrP) (1-36), and abaloparatide (ABL) have the same effect on *Crem*, *Epn3*, *Hdac4*, *Ppp2r2a*, and *Dvl3* messenger RNA (mRNA) expression. Primary differentiating calvarial osteoblasts were treated with 1 nM of PTH (1-34), PTHrP (1-36), and ABL for 4 hours prior to harvest, followed by quantitative reverse-transcription polymerase chain reaction for *Crem*, *Epn3*, *Hdac4*, *Ppp2r2a*, and *Dvl3* mRNAs. All data are expressed relative to the housekeeping gene *β-actin* and represent mean ± SD of n = 3 independent experiments. *P less than .05; **P less than .01; ***P less than .001; ns, not significantly different.

qRT-PCR data showed that *Crem*, *Epn3*, *Hdac4*, *Ppp2r2a*, and *Dvl3* mRNAs were similarly regulated by all 3 peptides even if not all were significantly so (Fig 5), confirming the RNA-seq data (see Table 1).

**Figure 6.**
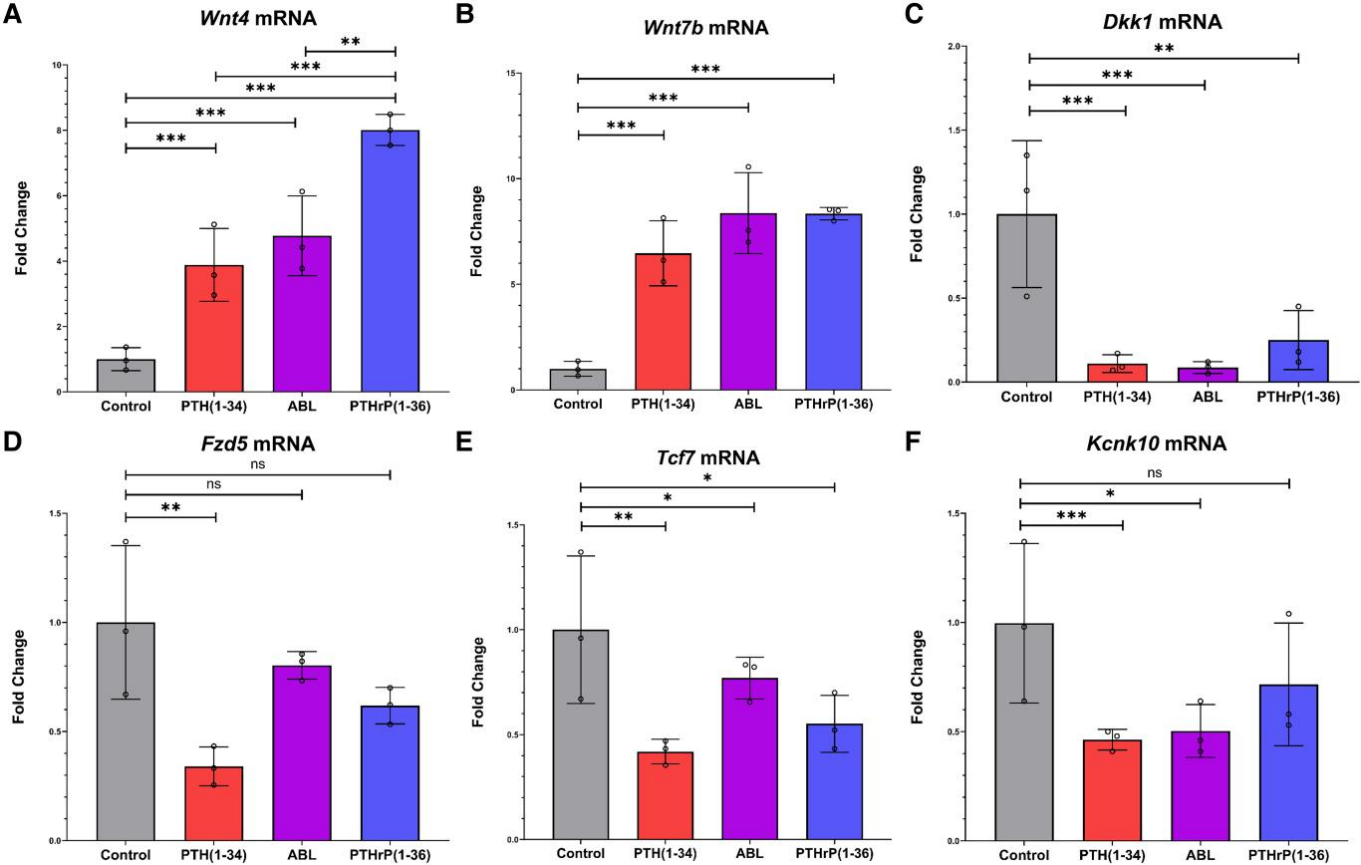
Parathyroid hormone (PTH) (1-34), PTH-related protein (PTHrP) (1-36), and abaloparatide (ABL) regulation of *Wnt4*, *Wnt7b*, *Dkk1*, *Fzd5*, *Tcf7*, and *Kcnk10* messenger RNAs (mRNAs). Primary differentiating calvarial osteoblasts were treated with 1 nM peptides for 4 hours, followed by quantitative reverse-transcription polymerase chain reaction for *Wnt4*, *Wnt7b*, *Dkk1*, *Fzd5*, *Tcf7*, and *Kcnk10*. All data are expressed relative to the housekeeping gene *Rpl13a* and represent the mean ± SD of n = 3 independent experiments. *P less than .05; **P less than .01; ***P less than .001; ns, not significantly different.

Further qPCR analyses show additional mRNA response patterns that mimic their respective RNA-seq data bar one, *Kcnk10* (see Table 1). *Wnt4* and *Wnt7b* are both upregulated by all 3 peptides compared to control, and it is very noticeable that PTHrP yields a significantly greater stimulation of *Wnt4* than the other peptides. *Dkk1*, *Fzd5*, and *Tcf7* are all downregulated by all 3 peptides (Fig 6). These qPCR data analyses show all 3 peptides downregulated *Kcnk10*, which differs from the RNA-seq data showing upregulation for all 3 peptides, PTH (1-34) having the greatest effect of them all.

### Examination of Genes of Interest With Small Interfering RNA Knockdowns of SIK1, SIK2, SIK3, CRTC1, CRTC2, and CRTC3

Since several of the genes followed the same differential regulation by these peptides as *Rankl*, we investigated if the genes were regulated through the SIK/CRTC pathway, as *Rankl* is [13]. We and others have shown that PTH inhibits SIK2 and 3 resulting in dephosphorylation of CRTC2 and 3, the translocation of the latter into the nucleus, and a resultant increase in *Rankl* transcription [13, 23].

**Figure 7.**
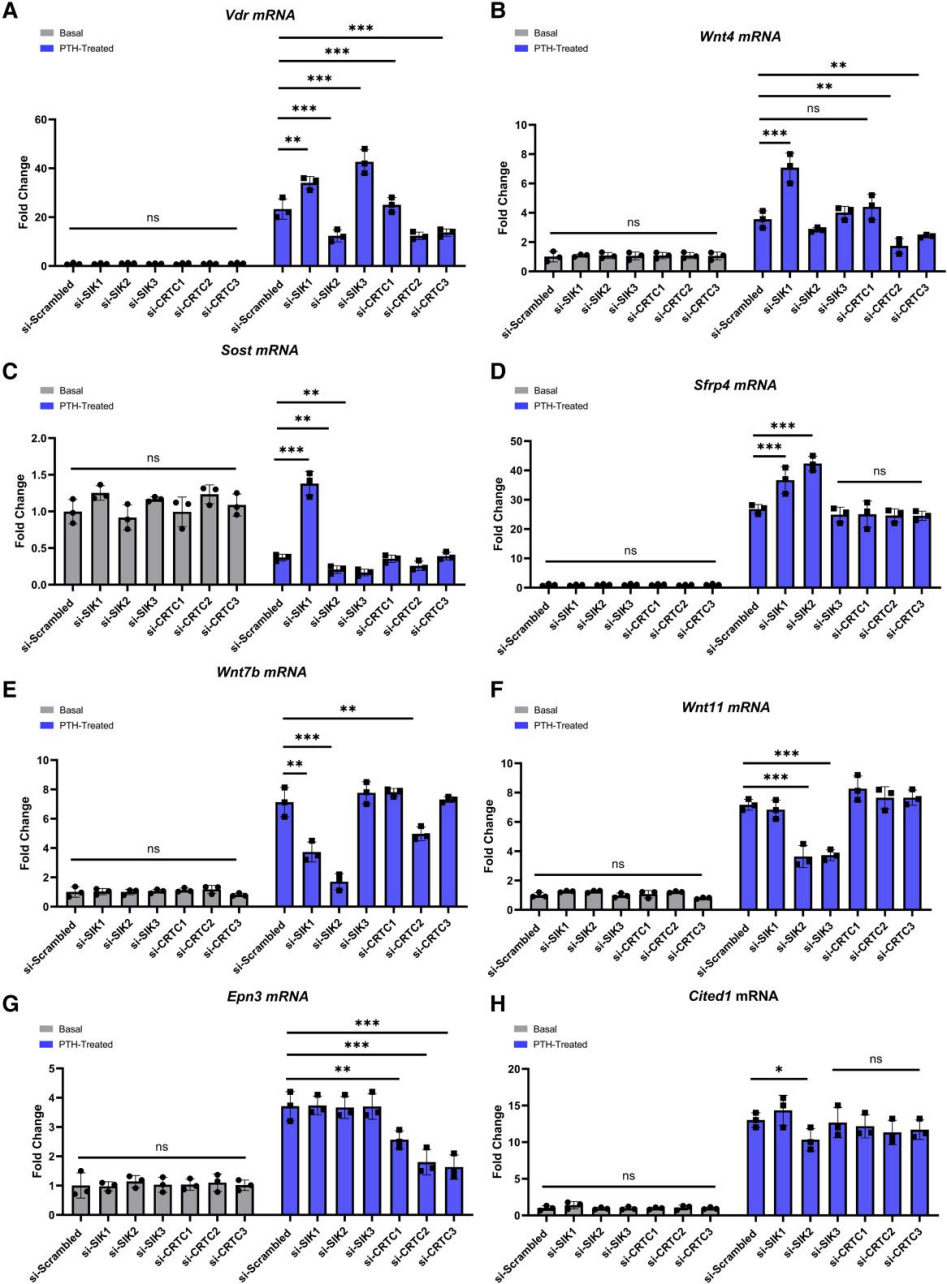
Role of SIK1/2/3 and CRTC1/2/3 in parathyroid hormone (PTH) (1-34) regulation of *Vdr*, *Wnt4*, *Sost*, *Sfrp4*, *Wnt7b*, *Wnt11*, *Epn3*, and *Cited1* messenger RNA (mRNA) in mouse calvarial osteoblasts. Primary mouse calvarial osteoblasts were cultured to 70% to 80% confluence. The cells were given scrambled or small interfering RNAs (siRNAs) for si-*Sik1*, si-*Sik2*, si-*Sik3*, si-*Crtc1*, si-*Crtc2*, or si-*Crtc3* (40 pmol) in lipofectamine RNAiMAX for 48 hours in osteoblast-differentiating medium. The cells were then treated with or without PTH (1-34) at 10 nM for 4 hours; RNA was harvested to determine mRNA in basal and PTH-treated samples compared to scrambled controls. All data are expressed relative to the housekeeping gene *Rpl13a* and represent mean ± SD. *P less than .05; **P less than .01; ***P less than .001; ns, not significantly different.

*Cited1*, *Vdr*, *Wnt11*, *Wnt4*, *Wnt7b*, *Sfrp4*, *Epn3*, and *Sost* mRNA abundance were examined after SIK1/2/3 and CRTC1/2/3 were knocked down in osteoblastic cells that were then treated with PTH (1-34) (Fig 7). The knockdowns were effective as seen by 70% to 80% decreases in *Sik* and *Crtc* mRNA expression and 60% to 95% decreases in their proteins (data not shown; under consideration in a separate manuscript). Knockdowns did not significantly change any of the aforementioned mRNAs under basal conditions but substantial individual/combined regulation was found in these samples when compared to scrambled controls and PTH (1-34) treatment. Analysis of the effects of SIK1/2/3 or CRTC1/2/3 knockdowns revealed complex relationships; data show that some of these genes are regulated by both SIKs and CRTCs (*Vdr*, *Wnt4*) as *Rankl* is, while others (*Sfrp4*) are regulated by SIKs but not CRTCs, in the same way as *Sost* is and some require SIK expression [23].

*Vdr* mRNA significantly increased with PTH treatment and SIK1 and SIK3 knockdowns when compared with scrambled controls; *Vdr* mRNA levels significantly decreased with knockdown of SIK2 and CRTC2/3 compared with scrambled controls. Knockdown of CRTC1 had no significant effect. Similarly, *Wnt4* mRNA significantly increased with PTH treatment and SIK1 knockdown when compared with scrambled controls; *Wnt4* mRNA significantly decreased with CRTC2/3 knockdown compared with scrambled controls. SIK2/3 and CRTC1 knockdowns had no statistically significant effects.

*Sost* mRNA significantly increased with SIK1 knockdown compared with scrambled controls; *Sost* mRNA significantly decreased with SIK2/3 knockdowns compared with scrambled controls. Knockdowns of CRTC1/2/3 had no statistically significant effects. *Sfrp4* mRNA significantly increased in SIK1/2 knockdowns when compared with scrambled controls; SIK3 and CRTC1/2/3 had no statistically significant effects.

*Wnt7b* mRNA significantly decreased with SIK1/2 and CRTC2 knockdowns compared with scrambled controls. SIK3 and CRTC1/3 knockdowns had no statistically significant effects. *Wnt11* mRNA significantly decreased with SIK2 and SIK3 knockdown when compared with scrambled controls; SIK1 and CRTC1/2/3 knockdowns had no statistically significant effects.

*Epn3* mRNA significantly decreased with CRTC2/3 knockdown compared with scrambled controls. SIK1/2/3 and CRTC1 knockdown had no statistically significant effects.

*Cited1* mRNA was barely affected by any of the SIK or CRTC knockdowns, suggesting it is not regulated by PTHR1 through this pathway.

## Discussion

In this study we show that PTH (1-34), PTHrP (1-36), and ABL treatment of calvarial osteoblasts in vitro result in quantitatively differing effects on the osteoblast transcriptome using RNA-Seq, GO, and additional qRT-PCR of a number of genes of interest but qualitatively are similar. We further investigated the mechanism of regulation of some of these genes using SIK1/2/3 and CRTC1/2/3 knockdowns in cells treated with PTH (1-34) and found several commonly regulated by SIK or CRTC-dependent pathways while others showed complex regulation.

RNA-Seq data revealed PTH (1-34) regulated the most genes (367), followed by ABL (179 genes) and then by PTHrP (1-36) (116 genes), with PTH (1-34) generally having the greatest fold effects but with 83 genes shared by all 3 peptides. GO analyses show biological processes are similar between the 3 peptides but have some pathway-specific differences. All 3 peptides regulated genes involved in ossification, cAMP signaling, and epithelial cell proliferation, while PTH (1-34) and PTHrP (1-36), but not ABL, regulated genes of branching morphogenesis by GO. The latter may reflect the role of PTHrP in mammary gland development [24]. PTH (1-34) and ABL show an almost identical pattern otherwise, including Wnt signaling, hormone transport and secretion, bone mineralization, and actin filament bundle organization. PTHrP (1-36) regulates these pathways differently when compared with either of these peptides. These similarities in pathway expression among PTH (1-34) and ABL may explain why they have similar effects in vivo and why PTHrP (1-36), despite being an analogue of ABL, did not have the same anabolic effects on bone mineral density [25]. With respect to molecular functions and cell components, PTH (1-34) gave the greatest and most significant effects, while all 3 peptides regulated phosphoric ester hydrolase activity (a feedback control for cAMP action), G protein–coupled peptide receptor activity, and nuclear and neuropeptide receptor activity, many of which were reflected in the cell component designation of receptor complex, which was significant for PTH (1-34) and ABL.

Based on the RNA-Seq data, we selected a handful of genes to confirm several notable expression patterns elicited by these peptides both in the RNA-Seq data and qPCR. The first pattern of note which we found both in the qPCR and RNA-Seq data is that *Vdr*, *Cited1*, *Pde10a*, *Wnt11*, and *Sfrp4* followed the reported pattern of expression profiling of *Rankl* in response to these peptides: PTH (1-34) produced the greatest increase in expression of these genes compared with control, while PTHrP (1-36) and ABL resulted in a moderate increase in gene expression compared with control (see Fig 5).

A previous report showed that daily administration of PTH (1-34) for 48 hours decreased renal *Vdr* mRNA expression by 15% in wild-type mice [26]. However, our data show that *Vdr* mRNA is increased by PTH (1-34) in osteoblasts and this may be explained by the periodicity of treatment and cell type. In fact, one group showed that PTH1R was present in mandibular/alveolar mouse bone at the earliest stages examined in embryogenesis while the VDR appeared only later with maximal expression at E18, implying it may be induced by PTH or PTHrP action [27]. In a prehypertrophic chondrocyte cell line, PTHrP was shown to substantially increase *Vdr* expression and the authors suggested there was a functional paracrine feedback loop modulating chondrocyte differentiation [28]. In our case, the highest regulation by PTH (1-34) in osteoblastic cells intimates that this hormone may control 1,25(OH)_2_ vitamin D_3_ action on these cells.

The second gene showing the same pattern of regulation as *Rankl*, *Cited1*, has also been reported to be upregulated significantly by PTH (1-34); Wt9 osteoblastic cells showed maximal upregulation of *Cited1* after 4 hours of PTH treatment that was blocked by PKA inhibition [29]. They also observed that in calvarial osteoblasts derived from *Cited1* knockout mice treated intermittently with a cAMP-selective analogue of PTH, [G1, R19]hPTH (1-28), there were greater increases in mineralization, which identifies CITED1 as a negative regulator of osteoblast differentiation. The protein is a transcriptional coactivator of the CBP/p300-mediated transcription complex that interacts with Smads. Its upregulation by PTH (1-34) may be a feedback control.

The general function of phosphodiesterases is to hydrolyze cAMP and cyclic GMP second-messenger molecules, thus regulating them as second messengers [30]. PDE10A has the highest affinity for cAMP, and most of our knowledge on it comes from studies in the brain [30, 31]. However, a recent study examined *PDE10A* expression in bone marrow-derived mesenchymal stromal cells isolated from a patient cohort undergoing hip replacement therapy [30]. They report that *PDE10A* is upregulated in response to mechanotransduction, and that its upregulation impairs osteogenic signals and that an increase in cAMP was the key driver in the observed results. Our data show that *Pde10a* mRNA is upregulated significantly by all 3 peptides but with PTH (1-34) showing by far the largest increase in its expression. It is most likely that this is feedback regulation to control cAMP concentrations, and the highest induction by PTH (1-34) reflects the highest cAMP produced by this peptide in these cells.

SFRP4 has been shown to have deleterious effects on osteoblasts/osteocytes. Secreted frizzled–related proteins (SFRPs) are well-known antagonists of Wnt family signaling by directly binding the Wnts as decoy receptors or by forming nonfunctional Wnt complexes via Frizzled (Fz) proteins [32]. Canonical Wnt signaling typically promotes bone formation so any complex that removes or inhibits its function is likely to negatively affect osteoanabolic processes, as shown in a study using transgenic mice overexpressing SFRP4; the researchers found that they exhibited comparatively lower bone mass [33]. Our data show that *Sfrp4* also has the highest upregulation in response to PTH (1-34) when compared to ABL and PTHrP (1-36) and may also be a feedback-control mechanism in response to cAMP levels. The greater effect of PTH (1-34) may mean greater restraint on Wnt action in the osteoblast compared to ABL.

Our pathway analyses and subsequent qPCR show that *Wnt4*, *Wnt7b*, and *Wnt11* all have significantly increased mRNA expression in response to all 3 peptide treatments compared to control but do not do so with the same pattern. *Wnt11* mimics the *Rankl* expression profile with PTH (1-34) having by far the greatest effect compared with ABL and PTHrP (1-36), while *Wnt4*/*Wnt7b* expression was highest with PTHrP (1-36) and PTH (1-34) having a lesser effect. WNT4 was known to be a noncanonical WNT family member but has been shown to act by both canonical and noncanonical pathways and to have a number of roles in bone [34]. WNT11 appears to stimulate osteogenesis through the canonical pathway, and WNT7B seems to also act through the canonical pathway to regulate limb development [35, 36].

The effects of PTH (1-34) and its analogues on the osteoblast transcriptome illuminate the exquisite nature of bone modeling and remodeling. Perhaps choosing an earlier time point would yield different results, particularly if performed to capture immediate early genes. Nonetheless, *Rankl*, *Vdr*, *Cited1*, *Pde10a*, *Sfrp4*, and *Wnt11*, which were differentially regulated in a similar pattern by the 3 peptides, suggested that these are affected by the initial differences in cAMP/PKA signaling and may also involve the SIK/CRTC/bZIP pathway, which regulates *Rankl* transcription [13]. Conversely, it is possible that the genes that were similarly regulated by all 3 peptides are governed by the SIK/HDAC pathway as shown by the similar regulation of *Sost* and *Mmp13*, both of which are controlled through the SIK/HDAC arm. qRT-PCR analysis of these genes in calvarial osteoblasts treated with 8-bromo-cAMP, myr-PKI, si-*Sik*s, si-*Crtc*s, or si-*Hdac*s would be able to confirm this hypothesis [13].

Knockdown of SIK1/2/3 and CRTC1/2/3 followed by treatment with and without PTH (1-34) (see Fig 7) showed that *Vdr* mRNA levels were significantly increased, compared with PTH (1-34) treatment, in response to si-*Sik1* and si-*Sik3* and decreased with CRTC2/3 knockdown similar to our observations with *Rankl* [13 and unpublished data]. *Wnt4* mRNA levels were also similarly affected by SIK1 and CRTC2/3 knockdown, suggesting that these 3 genes (*Vdr*, *Rankl*, and *Wnt4*) are regulated through the same pathway.

*Sost* and *Sfrp4* (both WNT pathway inhibitors) seem to be controlled similarly, by SIKs but not CRTCs, possibly both through the type II HDAC arm, although this would need to be proven for *Sfrp4*. Notably, PTH (1-34) decreases *Sost* expression, which has been thought to be a major part of its anabolic effects on bone, while it increases *Sfrp4* expression, which seems to be a feedback control of the WNT pathway.

PTH (1-34) stimulation of *Wnt7b* and *Wnt11* mRNA levels was significantly decreased by both SIK1/2 and CRTC2 knockdown. This suggests a positive role for SIK action in *Wnt7b* and *Wnt11* transcription. A similar observation has been made for transforming growth factor-β–stimulated transcription of *PAI-1* [37], and the authors speculated that the SIKs were involved with required phosphorylation of coactivators binding to *P*-Smads in the nucleus, but this is an open question, as it is for how SIKs might regulate *Wnt7b* and *Wnt11* transcription by osteoblasts.

It is notable that *Cited1* transcription is not regulated by the SIKs, and this was observed previously, indicating that there are a number of genes that PTH controls independently of this pathway [23]. It is possible that these are directly regulated by PKA phosphorylation of CREB and do not involve the CRTCs or by some other unknown pathway.

Limitations of this study include the fact that while changes in gene expression were validated by RT-PCR, none were validated at the protein level. In fact, phospho-proteomics (not just proteomics) would be valuable to complement the RNA-seq data in the future. As noted earlier, there is also the limitation that only 1 time point was chosen, 4 hours of treatment (although this is a time point when we have found changes in many osteoblastic genes [16]) and only 1 dose was used. A higher dose may show that ABL and PTHrP (1-36) have the same effect at this time point as suggested by observations with *Rankl* expression [13]. Another limitation is the use of rat PTH (1-34) in the present study and not human PTH (1-34), that is, teriparatide, which could yield some differences, yet we have found that both forms of PTH (1-34) generate the same stimulation of cAMP with osteoblastic cells (data not shown).

The findings in this study highlight the complexity of the genetic and functional events that are triggered by PTH (1-34) and its analogues. Although we discovered many genes that seemingly fit into known paradigms, there were many genes that should be further evaluated to understand the importance of PTHR1 and how it affects downstream signaling. A closer examination of some of these genes might reveal intricacies in the interactions of PTHR1 and PTH-derived treatments just as further delineation of which events are attributable to signaling mechanisms triggered by PTH (1-34), PTHrP (1-36), and ABL would allow for further refining of future treatments for osteoporosis.

## Data Availability

Some of the data sets generated and/or analyzed during the current study are not publicly available but are available from the corresponding author on reasonable request.

## Abbreviations

ABL: abaloparatide
cAMP: cyclic adenosine monophosphate
CRTC: CREB-regulated transcriptional coactivator
FC: fold change
FDR: false discovery rate
GO: gene ontology
mRNA: messenger RNA
PKA: protein kinase A
PTH: parathyroid hormone
PTHrP: parathyroid hormone–related protein
qRT-PCR: quantitative reverse-transcription polymerase chain reaction
RNA-Seq: RNA sequencing
SFRPs: secreted frizzled–related proteins
SIK: salt-inducible kinase
siRNA: small interfering RNA
